# Co-Evolution of Complex Network Public Goods Game under the Edges Rules

**DOI:** 10.3390/e22020199

**Published:** 2020-02-08

**Authors:** Xingping Sun, Yibing Li, Hongwei Kang, Yong Shen, Jian Peng, Haoyu Wang, Qingyi Chen

**Affiliations:** School of Software, Yunnan University, Kunming 650000, China; sunxp@ynu.edu.cn (X.S.); Ybing_Li@163.com (Y.L.); ynujianpeng@foxmail.com (J.P.); why@mail.ynu.edu.cn (H.W.); hexen@163.com (Q.C.)

**Keywords:** cooperation, public goods game, co-evolution, network, entropy

## Abstract

The reconnection of broken edges is an effective way to avoid drawback for the commons in past studies. Inspired by this, we proposed a public goods game model under the edges rules, where we evaluate the weight of edges by their nodes’ payoff. The results proved that the game obtains a larger range of cooperation with a small gain factor by this proposed model by consulting Monte Carlo simulations (MCS) and real experiments. Furthermore, as the following the course of game and discussing the reason of cooperation, in the research, we found that the distribution entropy of the excess average degree is able to embody and predict the presence of cooperation.

## 1. Introduction

The emergence of cooperative behavior in rational selfish groups remains an interdisciplinary problem. The game of public goods [1,2,3] is a standard metaphor for social dilemmas. Cooperation in group games has received extensive attention, from family relationships to global warming and other human behaviors. Selfish players pursue maximization of personal interests at the expense of cooperator’s, leading to drawbacks for the commons [4]. To avoid such harm, many mechanisms have been used to promote cooperation, such as punishment [5,6,7], reputation [8,9,10], reward [11,12,13], voluntary participation [14,15], social diversity [16,17,18], and migration [19,20,21].

In real life, many complex factors lead to changes in game outcomes, which evolve over time, so evolutionary games more accurately model real-world systems. Studies have shown that the co-evolution of network structure and strategy has profoundly affected the study of evolutionary games [22,23,24,25]. Zimmermann [26,27] examined the impact of the “re-edge reconnection” mechanism on cooperative evolution for the first time. They assumed that, if the player is not satisfied with the performance of the current opponent, they will cease their connection with a certain probability. The results showed that only a small probability of re-connection of the broken edge is needed, and the level of cooperation in the system can be significantly improved, but the calculation of node satisfaction is relatively simple. Santos [28] addressed the relationship between the two nodes in an extreme way: if player A’s strategy on one side is cooperative, then the other player B is satisfied with player A. Player B can choose to disconnect and randomly select new neighbors. They stated that, for players who only know local information, re-selecting the defector as a neighbor is a good choice. Pacheco et al. [29,30] considered the existence of active connections in the population. They assumed that the connections between players have different life cycles, and some equivalent evolutionary game dynamics can be used to describe the payoff matrix to some extent, which eventually makes natural selection favor cooperation. Verma [31] used the framework of evolutionary game theory to analyze the evolution of corruption and honesty strategies in structural populations that are characterized by complex interdependent networks, and discussed the influence of changes in network topology, average number of links, and asymmetry of population size of citizens and officials on the spread of bribery events. Hirofumi Takesue [32] studied a new prisoner game with punishment and exchange partner mechanisms. The researchers stated that the mobility of social relationships has a profound impact on maintaining cooperation.

In the past studies, although there are some researches on the promotion mechanism of disconnection and reconnection, most of them are based on the two-player game. There are few studies on edge disconnection and reconnection in multiplayer games, especially in public goods game. Additionally, most of the studies are rough about the calculation of the edge breaking conditions, and not fit enough for the real life. What is more, it is well known that topologies and strategies interact in co-evolution, and we want to understand the mystery of co-evolution. We wonder how the choices of nodes affect the topology of network and how the topology affects nodes’ choices in return. Is the influence of these different network topologies on the public goods game similar to the two-player game? We consider a new co-evolution mechanism of network structure and strategy (the co-evolution of network structure and strategy is called co-evolution in the rest of this paper) under edge rule, and explore the reason that brings up cooperation.

The next part of this paper is organized, as follows. In the second section, we introduce the related work and describe the game model under edges rules in detail. In the third section, we present the Monte Carlo simulation results and actual observer data. In the last section, we provide the conclusion of this paper.

## 2. Materials and Methods

### 2.1. Related Work

The theory of complex network is an interdisciplinary subject with rapid development. However, thus far, there has been no certain definition of complex network in the research. The complex network is not defined precisely and strictly. It can be regarded as a topological abstraction of a large number of real complex systems. It is neither a regular network nor a random network, but a network with different statistical characteristics. Graph *G* shows an ordered pair (*V, E*), where *V* is the Vertical Set and E is the Edges Set. They can also be denoted as *V*(*G*) and *E*(*G*). The graphs in the rest of this paper refer to undirected graphs.

#### 2.1.1. Regular Network

**Figure 1:**
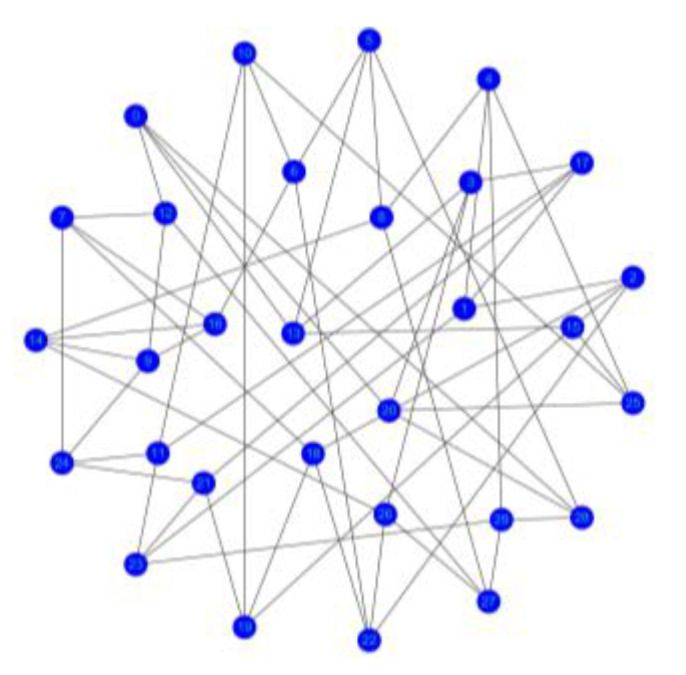
In the figure, the network is an example of regular network with 20 nodes and four degrees.

The definition of the complex network mentioned in this paper is a generalized one. Regular network is the simplest model of that. In Figure 1, each node in the network has the same degree; the average path length of the network is proportional to the network scale; the whole network can be regarded as a combination of many “sub-networks” with the same structure; such a network is called a regular network. Globally coupled network, nearest-neighbor coupled network, and star coupled network are common regular networks.

#### 2.1.2. Public Goods Game Based on Complex Network

Public goods game (PGG) is a multi-players game with two strategies, which often describes the cooperation dilemma of players in interactive conditions. In the game, players can choose to invest in the common pool (cooperate) or not (defect). The pool’s funds will have an increase in real life, the increasing extent is denoted as gain factor *r* (*r* > 1). Funds in the common pool will be given away to players averagely. Subsequently, the payoff of a player participate in a game is:

(1) $$P_{x}=\left\{ \begin{array}{ll} \frac{rn_{c}}{g}\;-1 & \;\mathrm{if}\;S_{x}=C\;\\ \frac{rn_{c}}{g} & \;\mathrm{if}\;S_{x}=D \end{array}\right.$$

where *r* is the gain factor, *n_c_* is the number of cooperators in the group, *g* is the player number of the group, and *S_x_* is player *x*’s strategy; subsequently, *P_x_* represent the *x*-centered payoff. Nodes connected by edge are called neighbors of each other. Each player only plays with the neighbors. We define this player and his/her neighbors as a group, where there is a common pool. For each player, in each round of the game, cooperators incur more cost *c* than defectors. There are two extreme cases, one is whole network defect and the other is whole network cooperation. For the whole network defect, none of the players will receive any payoff in the game. Furthermore, players will receive the highest payoff for the whole network cooperation. 

Based on complex netwok, in the public goods game, the nodes in the network represent the players of the game, whereas the edges represent the interactive relationships between the players. In one round of the game, the payoff of a player not only comes from the self-centric pools, but also the neighbours-centric ones, thus the number of common pools that contributes to a player’s payoff is its degree plus one. A certain node chooses a strategy and the corresponding payoff is called one-time game. The completion of one-time game for every nodes in the network is a round of game.

#### 2.1.3. Analysis of Static Cumulative Payoff of Public Goods Game

There are two sources of the cumulative payoff of player *x* in a single game: (1) the revenue from the game centered on player *x*, being expressed as *P_x_*; (2) the payoffs from the game centered on *M_x_* neighbor players in which player *x* participates, expressed as *PN_x_*, and *PN_(x)y_* is the payoffs from the game centered on neighbor *y* in which *x* participated. If player *x* has no neighbors, the return is 0 (we only discuss the case of *M_x_* > 0). For the writing convenience, one thing needs to mention is that for a player *x*, when calculating the payoff of game that centered on itself, we add subscript *x* to the variables that are involved; when calculating the payoff of game that centered on its neighbor *y*, we add subscript *(x)y* to the involved variables.

The total payoff of a single game for a player is the summation *π_x_* of payoff of each PGG group that it participates in:

(2) $$\pi_{x}=P_{x}+PN_{x}=P_{x}+\sum_{y=1}^{M_{y}}PN_{(x)y}$$

It can be seen from Equations (1) that, when a player participates in a single game, the cumulative payoff *π_x_c__* of choosing a cooperation strategy can be expressed as:

(3) $$\begin{array}{ll} \pi_{x_{c}} & =P_{x}+\sum_{y=1}^{M_{y}}PN_{(x)y}\\ \; & =\frac{rn_{cc_{x}}}{g_{x}}-1+\sum_{y=1}^{M_{y}}\left(\frac{rn_{cc_{\left(x\right)y}}}{g_{(x)y}}-1\right)\\ \; & =r(\frac{n_{cc_{x}}}{g_{x}}+\sum_{y=1}^{M_{y}}\frac{n_{cc_{\left(x\right)y}}}{g_{(x)y}})-(M_{x}+1) \end{array}$$

According to Equations (3) and (4), we can reach the same conclusion: when a player participates in a single game, and the cumulative payoff *π_x_d__* of choosing a defection strategy can be expressed, as follows:

(4) $$\pi_{x_{d}}=r(\frac{n_{cd_{x}}}{g_{x}}+\sum_{y=1}^{M_{y}}\frac{n_{cd}{}_{{}_{(x)y}}}{g_{(x)y}})$$

### 2.2. Co-Evolution of Public Goods Game under the Edges Rules Model

**Figure 2:**
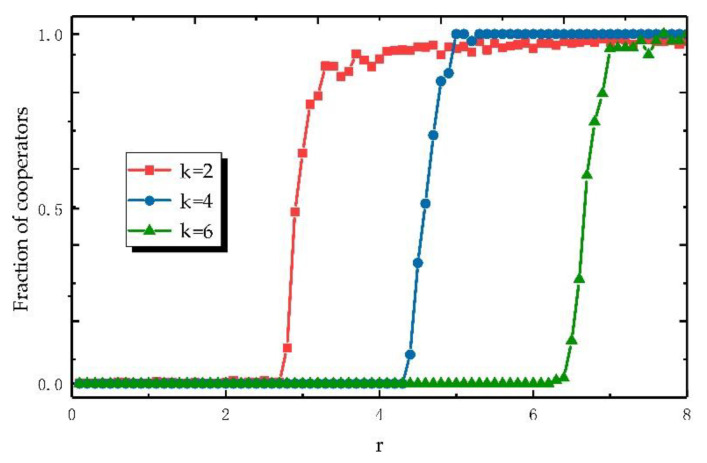
Under the rule without promotion mechanism, the fraction of cooperators changes with the gain factor *r* when the average degrees of nodes in the network are *k* = 2, *k* = 4, and *k* = 6.

We consider an evolving public goods game starting on a regular network with degree 4 (the choice of degree 4 refers to the square network commonly used in spatial evolution games [33]. Like what is shown in Figure 2, the selection of regular networks with different degrees has an impact on the experiment, but the conclusion is not affected). It is important to note that initial network is a regular network. With the advance of the game process, the degree of nodes is no longer regular. Additionally, the nodes in the network represent the players of the game, whereas the edges represent the interactive relationships between the players. 

Similar to Zimmermann et al. [25], we provide a co-evolution promotion mechanism under edge rules. The mechanism is divided into three stages: A. cut off the unsatisfactory connection. B. reconnection of dominant nodes. C. the isolated node rejoins the network. These stages are detailed, as follows.

#### 2.2.1. Edge Breaking Rule

**Figure 3:**
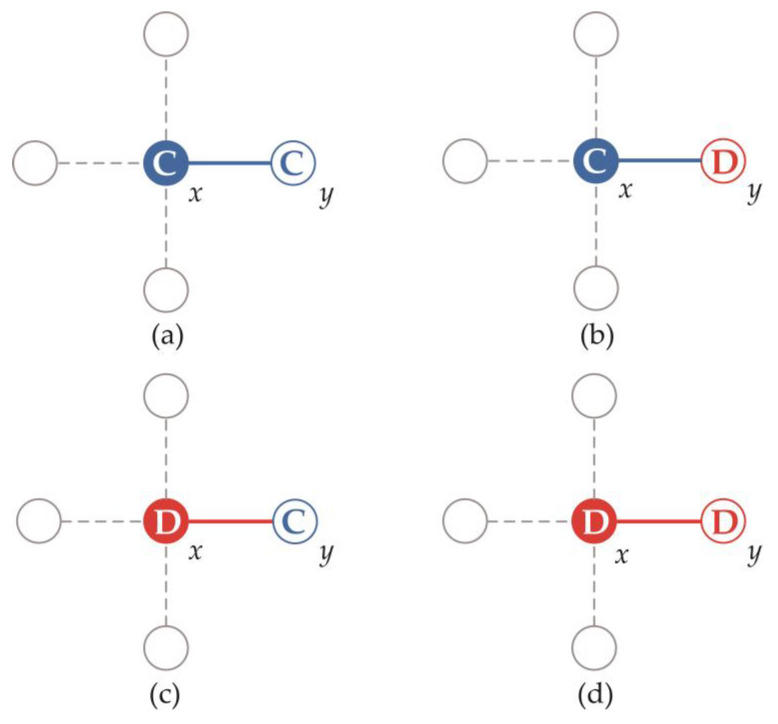
Figure shows four connection states in the game centered on x, from left to right and from top to bottom: (**a**) *C-C*, (**b**) *C-D*, (**c**) *D-C*, and (**d**) *D-D*. Blue indicates that the player’s game strategy is cooperation; Red indicates that the game strategy is defect; A solid circle indicates that the player is the center player, and a hollow circle indicates that is the neighbor player.

Before introducing edge rules, let us take a look at the nodes’ payoff. Nodes in the network have different connection states and strategy choices, which will bring different payoff to nodes. A node might have several edges in the network, here we focus on a certain edge of them. In Figure 3, we show four games that centered at *x*, where we focus on the unique solid edge of each subfigure.

For example, Figure 3a shows a *x*-centered game, where *x* and *y* are two cooperative nodes. The payoff of node *x* from edge *x-y* can be divided into two parts: (1) the cooperative node *y* invests *c* to the common pool, which will be averagely given to the players in the group after the effect with gain factor *r*. Thus, as a player in the group, node *x* gets *rc/g* here. (2) It takes *c* to choose cooperate for node *x*. The cost will also be apportioned to players in the group, thus the loss of node *x* is *c/g*. Overall, the payoff of node *x* from edge *x*–*y* is *rc/g − c/g*. Likewise, the payoff of node y from edge *x*–*y* can also be divided into two parts: (1) The funds of common pool n_c_ * c will be averagely given away to players in the group after the effect with gain factor *r*. Thus, node y gets (n_c_*c*r)/g here. (2) It takes *c* to choose cooperate for node *y*, thus the loss here is *c*. Overall, the payoff of node *y* from edge *x–y* is n_c_*c*r/g − c. In our model, the *c* is set as 1. Hence, we can obtain the results of other subfigures in Figure 3, which is shown in Table 1.

Three variables are involved in the edge breaking rule, as follows:

(1) Edge breaking value: the breaking value of an edge is the summation of its nodes’ payoffs.
(2) Edge breaking weight: the default edge breaking weight of an edge is 0. If edge breaking value is greater than or equal to 0, the breaking weight will minus 1, or else it will plus 3.
(3) Edge breaking probability: it is described by the cumulative Poisson distribution function, the primitive formula is following:

(5) $$\begin{array}{l} F=\sum_{m=0}^{weight}\frac{e^{-\lambda }\lambda^{m}}{m!}\;m\in N\wedge m\in [0,10]\\ weight\in N\wedge weight\in [0,10] \end{array}$$

where *F* represents the cumulative Poisson distribution function, *weight* represents the breaking-edge value of the node, *λ* represents the expectation, and *m* represents the number of events. In our model, we set the number of events *m* as 10 and the expectation *λ* as 6. These two specific values are back-reasoned by setting the breaking weight as 6 and the breaking probability as 0.5. For an edge, its breaking weight equaling 6 means that there are at least twice its breaking value is less than 0 in the game. The probability set as 0.5 means that once the former condition happens, the edge will be broken with half of probability.

#### 2.2.2. Edge Addition Rules

There are two conditions that leads to addition of edges in our model, they are shown as follows:

(1) Reconnection of isolated players. People in real life will not completely ignore the isolated players. Thus, the isolated players in our model are provided with opportunities to rejoin the game. Concretely, in a new round of the game, once an isolated player chooses to cooperate, it will be reconnected to the largest part of the network with the probability of 50%. It is noted that isolated nodes will not play in the game, unless it is reconnected to the network.
(2) New connections of dominant players. In graph theory, *G* is called a connected graph if any two nodes in an undirected graph *G* are reachable; otherwise, it is a unconnected graph. An unconnected graph is composed of two or more connected subgraphs, and these disjoint connected subgraphs become the connected components of the graph. The dominant nodes are the nodes whose strategy is cooperation and cumulative payoff is great than 0 in the largest connected component of the game network. Like what is happening in real life, dominant people are more willing to have favorable relations. Therefore, in our model, each dominant player can randomly select a player from the non-isolated cooperative ones to establish a connection.

The increment of edges should have a limitation given the resources it takes in real life. Briefly, we take the number of edges in the initial network as the maximum limitation. Edge addition rules will not function once the number of edges reaches its maximum.

#### 2.2.3. Dynamics of Game Player Strategy Adjustment

After each game, player *x* randomly selects a neighbor *y* from its neighbors to update its own strategy. We use the Fermi rule to describe the game dynamics [34] with the previous neighbors. Fermi rule, as the one of the simpler methods of information dissemination on the network, is often used in the study of public goods game: the probability *W_(Sx__→Sy)_* of player *x* adopting neighbor *y*’s strategy is related to the difference between their last payoff:

(6) $$W_{\left(S_{x}\to S_{y}\right)}=\frac{1}{1+\exp(-\frac{\pi_{y}-\pi_{x}}{k})}$$

where *π_x_*(*π_y_*) represents the payoff of the player *x*(*y*) and *k* is the noise coefficient, which indicated that there are still some non-completely rational players (the appearance of non-complete rationality lies in the lack of people’s control over their emotions and the limitation of their thinking rationality in real life). When *k* = 0, the player is completely rational; whether the strategy is updated completely depends on the difference in payoff. *k* > 0 represents a certain degree of randomness; that is, low-return node strategies will be learned by high-return players. As *k*→*∞*, the player policy update is completely random. All the values of *k* in this paper are 0.1. In the Monte Carlo simulations (MCS) (see Supplementary Material), the results of our edge rule co-evolution model are obtained on a regular network of 1000 nodes. We would like to stress that, even though regular network does not simulate the network structure of real systems very well, we would like to start with them, because simple topologies often allow for us to more clearly explore things. We carried out 100 Monte Carlo processes in order to make the game reaching a stationary state, and to ensure the accuracy, we carried on the repeated experiment for 1000 times in independent running states, and the data we showed in this paper is the average value of them. The node cannot change its strategy during the round of game.

## 3. Results

### 3.1. Preliminary Simulation Experiment

We chose a simple regular network as the key of our research to improve the clarity of our research and explore the nature of problem. Our research starts from the observation of game that has no promotion mechanisms. The results in Figure 2 show that the higher the average degree, the more difficult it is for regular network to produce cooperation, which makes us interested in the relationship between the degree of nodes and fraction of cooperators. At the same time, cooperative behaviors begin to occur when the gain factor approaches the average degree of the network under the premise that the initial game network is a regular network.

**Figure 4:**
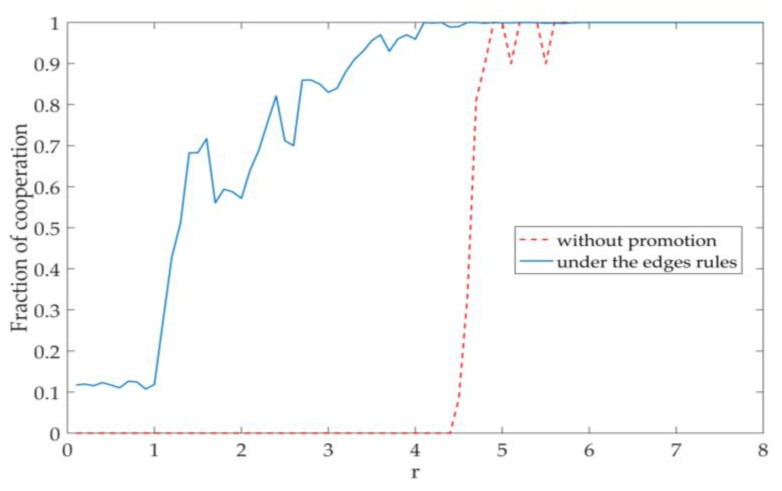
A comparison between the co-evolution mechanism of edge rules and the rule without promotion mechanism game model. The dotted line is the rule without promotion mechanism, and the solid line is the co-evolution rule under the edge rule.

We did a comparative experiment between a model under edges rules and one without any promotion mechanism based on the above analysis of network topology and cooperative behavior. From Figure 4, we can observe that the cooperation of the model under edges rules appears earlier than the other one (that without any promotion mechanism), and its cooperation is also more stable when the *r* value is low. What is more, the convergence speed of the model under edges rules is faster than the one without promotion mechanisms. We want to know what causes all of these changes, what kind of network topology can promote cooperation to the greatest extent without changing the gain factor, and what kind of co-evolution mechanism can promote the optimization of network topology. It should be noted that, in the Preliminary experiment, the game was simulated on a rule network of 100 nodes.

### 3.2. Simulation Analysis of Coevolution Model under the Edges Rules

**Figure 5:**
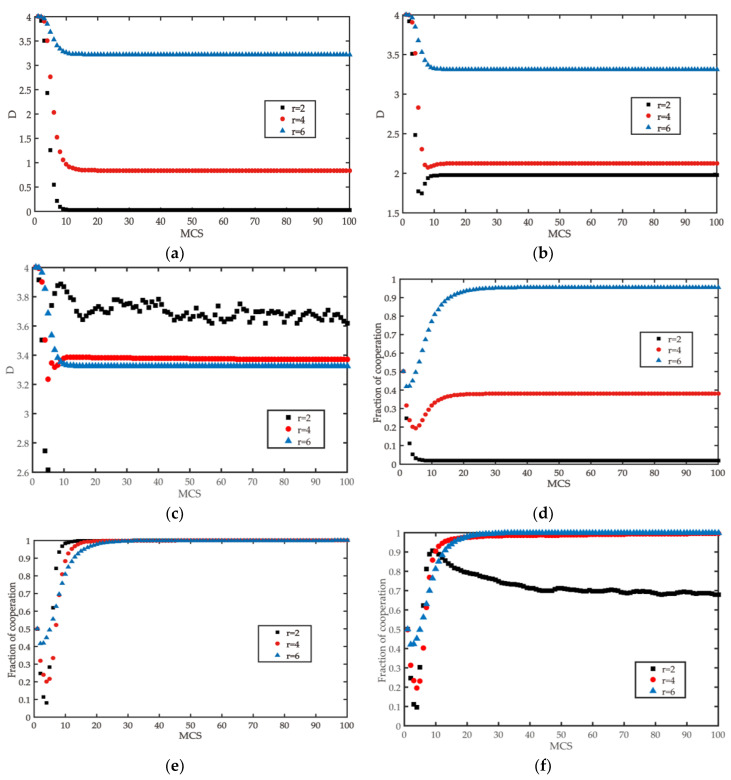
When the gain factor *r* = 2, *r* = 4, *r* = 6, the change of average fraction of cooperation and average degree under the edge rule game. (**a**,**d**) are the rules of edge breaking, (**b**,**e**) are the rules of advantage node and isolated node reconnection after edge breaking, and (**c**,**f**) are the rules of advantage node reconnection after edge breaking.

We will analyze the three stages of edge break reconnection in sections, and show the results in Figure 5. 

Under the edge breaking mechanism, as in Figure 5a, we can conclude that high gain factor brings high satisfaction, low willingness of a player to break the edge, and high network average degree; on the contrary, when the gain factor is low, the average degree of the network (the average degree of all nodes in the network) quickly declines, and the satisfaction of player to neighbor is obviously lower. Figure 5d shows that, under the edge breaking mechanism, in the very short process of MCS, defectors tend to increase, but the evolution results start to change after that stage. Soon there were cooperators at *r* = 2, although in a small number. It can be concluded that, when *r* > 4, the cooperators have better performance. The reason for this phenomenon is that the edge breaking mechanism decreases the average degree of the initial network, which makes the defectors have a rebound trend. However, with the evolution of network topology and the increase of the number of games, cooperation appears in the network when the gain factor is very small. In the long run, breaking the connection with low satisfaction promotes the emergence and evolution of the cooperation behavior of public goods game in the regular network. 

The reconnection mechanism of isolated nodes makes the network have more connections when the gain factor is small. We can know that it will bring a certain degree of confusion to the cooperative behavior in the early stage of network. However, the network cooperation will soon be realized under such a condition. Thus, conclusions can be drawn that the mechanism of isolated node reconnection greatly promotes the evolution of cooperation. 

The reconnection mechanism of the dominant node and isolated node makes the average degree of the nodes make stable quickly in the network. However, the mechanism of dominant node reconnection weakens the promoting effect of isolated nodes’ reconnection on cooperative behavior. When *r* = 2, a fraction of cooperation is near 0.68. The reason why we retain the dominant node mechanism is that dominant people are more willing to have favorable relations in real life and the average degree of the network is stable near *k* = 3.5 when *r* = 2, 4, 6, which gives it practical significance. 

### 3.3. The Relation between the Distribution Entropy of Excess Average Degree and Node Degree

**Figure 6:**
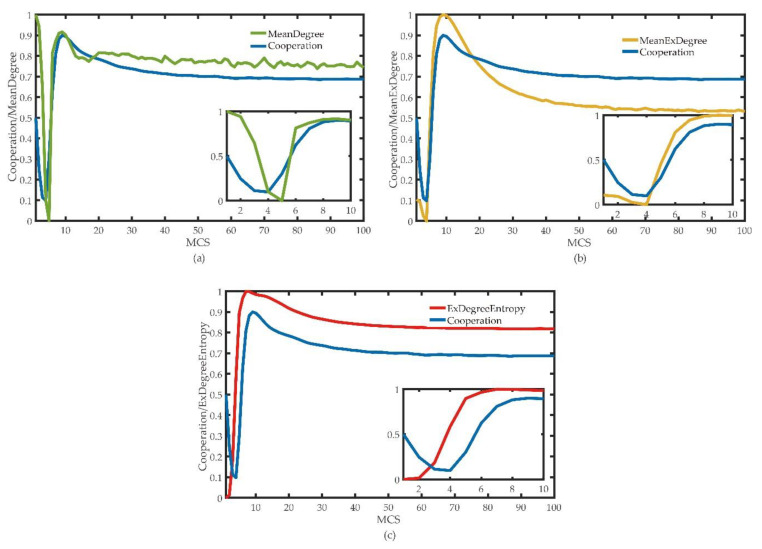
Under the edge rule, (**a**) the contrast between normalized average degree and fraction of cooperation, (**b**) the contrast between normalized excess average degree and fraction of cooperation, and (**c**) the contrast between the distribution entropy of excess average degree and fraction of cooperation *r* = 2. In order to show the results more clearly, we enlarged the fraction of cooperation and the comparison of the three values in the first 10 Monte Carlo simulation (MCS) processes, and displayed the results in the small figure in the lower right corner of each figure.

In Figure 5, we can observe that there is a close relationship between the degree and cooperative behavior in the game. Therefore, we compare the average degree and the fraction of cooperators to explore the relationship between them. However, the result of Figure 6a is that the average degree is directly proportional to the density of partners, which is contrary to the previous analysis. The average degree cannot explain the behavior of players well, and it cannot represent the characteristics of topology in the game. We suppose that the interaction between the group nodes affects the results, which is where the excess of average degree comes into our mind.

Suppose a node *x* and its degree is *D_x_*. Its excess average degree is the average degree <*D_nn_*> *_x_* of its neighbor:

(7) $$<D_{nn}{>}_{x}=\frac{1}{D}\sum_{j=1}^{D_{x}}D_{x_{j}}$$

We replace the excess average degree with the average degree and obtain the results of Figure 6b. The excess average degree can reflect the trend of the density of cooperators in the late stage of the game, but not in the early stage. It can be seen that the average degree and excess average degree are both means. The mean value often cannot represent the data characteristics when there are extreme values in the data.

Entropy is a measurement of the degree of system chaos that can indicate whether the state of an object is stable. Complex networks also have a similar definition of structure entropy to measure their heterogeneity. Based on this, we introduce the distribution entropy of excess average degree to study the relationship between the degree of nodes and cooperative behavior. We called it ‘the distribution entropy of excess average degree’, which is defined as:

(8) $$H_{nn}=\sum_{D_{nn}=1}^{N-1}(p\left(\langle D_{nn}\rangle \right)\log(p\left(\langle D_{nn}\rangle \right))$$

where *N* is the number of nodes in the network and *P*(<*D_nn_*>) is the probability that the excess average degree of nodes is <*D_nn_*>. For the convenience of calculation, the probability value in Equation (8) is set as:

(9) $$p\left(\langle D_{nn}\rangle \right)=\frac{\left[<D_{nn}{>}_{x}\right]}{\sum_{x=1}^{N}\left[<D_{nn}{>}_{x}\right]}$$

From Figure 6c, we can know that the distribution entropy of the excess average degree reflects the change of the fraction of cooperation in the co-evolution public goods game model under the edge rule well. In the early stage of he game, we observed that the change of the entropy of the distribution of the excess average degree is ahead of the fraction of the cooperators, which also verifies our assumption. In fact that the change of the network topology leads to the change of the fraction of cooperation. The topology structure changes first, and the behavior of the partners change accordingly. The average degree, the excess average degree and distribution entropy of the excess average degree are normalized in order to make the result clearer, as follows:

(10) $$\bar{E}=\frac{E-E_{\min}}{E_{\max}-E_{\min}}$$

### 3.4. A simulation Snapshot of the Co-Evolution under Edge Rules

**Figure 7:**
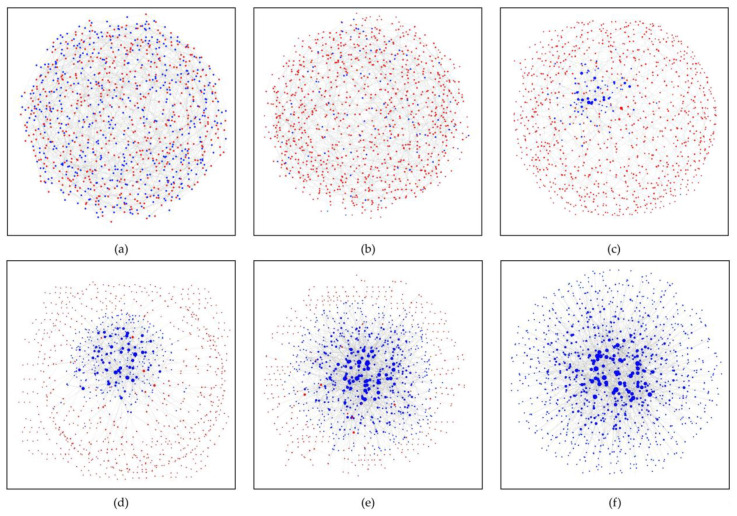
The topological structure change of the regular network with 1000 nodes caused by the evolution of edge rules. (**a**–**f**) are the 0, 2, 3, 4, 5, and 99 MCS processes, respectively. A red dot represents a defector, a blue dot a cooperator. k = 2, where the size of the node represents the size of the node degree.

We conducted 1000 repeated game experiments and selected a group of experimental data to show its topology. From the Figure 7a, the defector takes the upper hand (Figure 7a,b) in the early stage of the game. With the change of topology, there are more and more cooperators in the network (Figure 7c). With the proceeding of evolution, the number of cooperators in the network increases, and the connections of internal nodes increase (Figure 7d,e), and finally tend to be stable (Figure 7e,f). The fraction of cooperators in a game is proportional to the distribution entropy of excess average degree. It also has a certain predictability, which shows that the change of network structure has a profound impact on cooperation behavior. In addition, cooperation in small groups will contribute to large range of cooperation under the co-evolution of edge rules, and that is what we are expecting to see.

### 3.5. Actual Observer Data 

In the main body, we use Monte Carlo method to prove that the edge rule model promotes the generation and evolution of cooperative behavior. Here, we used data from real experiments to verify the simulation results (*r* = 2). We adopted the same real game experimental model as the simulated game model and recruited 30 volunteers in the School of Software of Yunnan University on 12 January 2020. 

The experiment is divided into two phases: the information-notifying phase and the decision-making phase. During the information-notifying phase, each volunteer will be informed of this information: the personal payoff of the previous round of the game, the strategy and payoff of each neighbor in the previous round, and the dominant nodes in the previous round. In the decision-making phase, corresponding to Monte Carlo simulation, volunteers need to make corresponding decisions that are based on gaining more personal payoff: strategies in a new round of games; whether to disconnect from neighbors and who are the neighbors with a broken edge; whether to increase the connection with the dominant node in the previous round and which dominant node to connect. For isolated volunteers, if they choose to cooperate in the new round, then they will have the opportunity to participate in the game again, otherwise they will not participate in the game. We ensure that each volunteer knows all of the rules before the experiment, and the volunteers complete the experiment anonymously on the computer throughout the process, to ensure the accuracy of the experiment.

**Figure 8:**
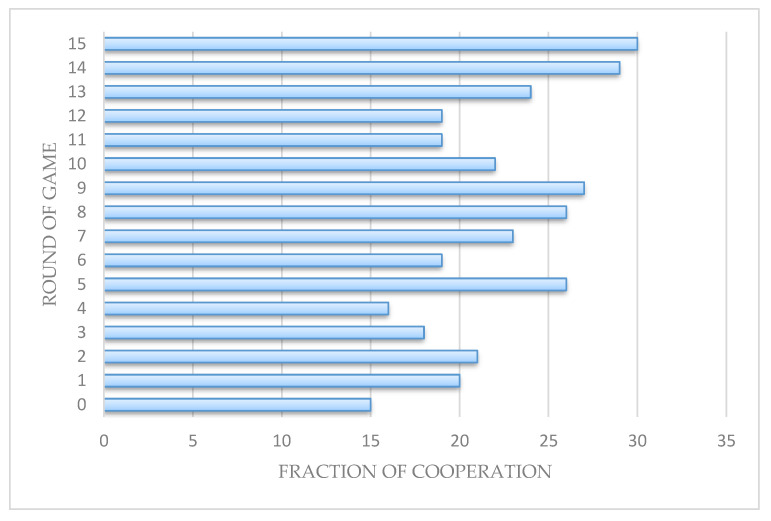
Under the edge rules, this is the fraction of cooperation of a real experiment. The *x*-coordinate is the fraction of cooperation, and the *y*-coordinate is round of game (*r* = 2).

**Figure 9:**
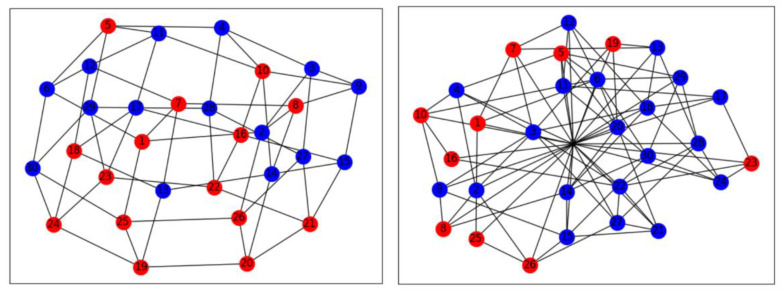

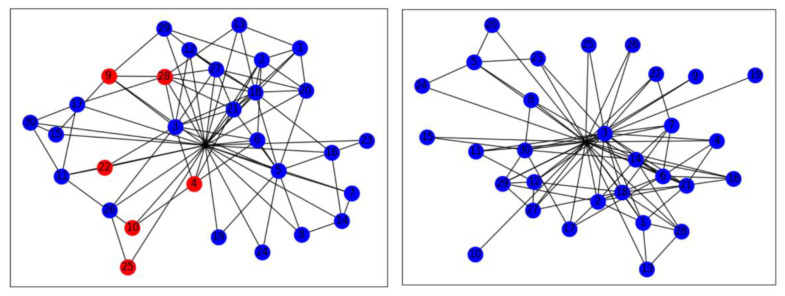
From top to bottom, and from left to right, the topological changes of the network in the 1st, 2nd, 7th, and 30th games in the real experiment and players’ strategy choices are shown in the figure (*r* = 2).

Figure 8 and Figure 9 shows the experimental results. The curve of fraction of cooperation in Figure 8 is similar to the simulated curve: there are some fluctuations in the early stage, and it eventually tends to be stable. This shows that both simulation results and real experimental results verify that the edges rules model promotes cooperation in the public goods game. The network topology in Figure 9 verifies the reason why the edge rules promote cooperation. It is the fluidity of connections in the network that makes cooperation nodes with more connections easy to appear in the network and promotes a wide range of cooperation behaviors, which is similar to the simulation results.

In general, the experiments show that the real data is similar to the simulated data, and the public goods model under edge rules promotes cooperation in the game of public goods.

## 4. Conclusions

When studying the public goods game based on complex networks, we get the following conclusions.

(1) The experimental results in this paper tell us that cooperative behaviors begin to occur when the gain factor approaches the average degree of the network under the premise that the initial game network is a regular network.

(2) We propose a co-evolution model of public goods game under the edges rules in order to achieve high cooperative behavior at low gain factors. The simulation results and real experiments show that the model can achieve a higher fraction of cooperative at a lower gain factor (*r* = 2). The reason for this phenomenon is that network evolution under the edges rules make more connected cooperation nodes appear in the game to promote the generation and evolution of cooperation.

(3) Under the premise of fixed gain factor (*r* = 2), the distribution entropy of the excess average degree can predict the turning point of fraction of cooperative, and fit the fraction of cooperative earlier and better when compared with the macroscopic indexes of network structure, such as average degree and excess average degree.

(4) For the solution of the ’drawback for common’, the usual punishment means is to use extra resources to find the free-riding behavior and implement the punishment. Our model does not deliberately design the evolution of network, under which players use their own judgment and behavior to complete the punishment (broken edges) and reward (added edges) in the pursuit of the maximization of their own interests, realizing the self-organization evolution of network structure. Our model provides a new idea for resolving the drawback for the common.

## Figures and Tables

**Table 1:** Unilateral static payoff analysis. In the table, when *x* and *y* choose different strategies in the game centered on *x*, the payoff of *x* and *y* are shown. Noted that it is one-time game payoff.

*x*–*y*	*x’*s Payoff	*y’*s Payoff
*C-C*	$\frac{r}{g}-\frac{1}{g}$	$\frac{rn_{c}}{g}-1$
*C-D*	$-\frac{1}{g}$	$\frac{rn_{c}}{g}$
*D-C*	$\frac{r}{g}$	$\frac{rn_{c}}{g}-1$
*D-D*	0	$\frac{rn_{c}}{g}$

## Supplementary Materials

The following are available online at https://www.mdpi.com/1099-4300/22/2/199/s1, the algorithm for the course of a simulation.
