# Supplementary material for: Co-Evolution of Complex Network Public Goods Game under the Edges Rules
Source: Entropy (Basel). 2020 Feb 8;22(2):199. doi: 10.3390/e22020199 (PMC7516628; doi:10.3390/e22020199)
Supplement: Supplementary file 1 [file entropy-22-00199-s001.pdf]

Supplement Materials

The algorithm for the course of a simulation

/*initialization*/

Create a regular network with N nodes, which is represented as an adjacency matrix adj.

Initialize node's strategy, and the strategy of node x is $strategy\_of\_node[x]$.

$F = \sum_{m=0}^{weight} \frac{e^{-\lambda}\lambda^m}{m!}$ $m \in N \wedge m \in [0,10], weight \in N \wedge weight \in [0,10]$// refer to Equation (5)

/*start game*/

For $gt = 1$ to $game\_times$

/*calculate node's payoff*/

For $x = 1$ to N

$n_c = GetCooperation\_Number(x)$

$g = GetGroup\_Number(x)$

$C\_payoff = \frac{rn_c}{g} - 1$ //refer to Equation (1)

$D\_payoff = \frac{rn_c}{g}$

If $S_x =$ 'C'

$P_x = P_x + C\_payoff$

Else

$P_x = P_x + D\_payoff$

End

/*calculate payoff from neighbors*/

For $y = 1$ to N

If $adj[x][y] = 1$ and $S_y =$ 'C'

$P_y = P_y + C\_payoff$ // refer to table 1

$Payoff\_from\_nb[y][x] = Payoff\_from\_nb[y][x] + C\_payoff$

If $S_x =$ 'C'

$Payoff\_from\_nb[x][y] = Payoff\_from\_nb[x][y] + (r-1)/g$

Else

$Payoff\_from\_nb[x][y] = Payoff\_from\_nb[x][y] + r/g$

End

End

If $adj[x][y] = 1$ and $S_y =$ 'D'

$P_y = P_y + D\_payoff$

$Payoff\_from\_nb[y][x] = Payoff\_from\_nb[y][x]_y + D\_payoff$

If $S_x =$ 'C'

$Payoff\_from\_nb[x][y] = Payoff\_from\_nb[x][y] - r/g$

End //according to table 1

End

End

End

/*update edge breaking weight*/

```
For i=1 to N
    For j=1 to N
        Calculate edge_breaking_weight[x][y]  //according to 2.2.1 (2) Edge breaking weight
    End
End
/*break edge according edge breaking weight */
For i=1 to N
    For j=1 to i
        temp = edge_breaking_weight[x][y]
        If edge_breaking_weight[x][y] > 10  then temp = 10  End
        If random_number < F[temp] then
            Break the connection with node x and node y
        End
    End
End
/* reconnection of isolated individuals */
Get the node list max_connected_component contained in the max connected component of the
current network
i = choice( max_connected_component )
For x = 1 to N
    If D_x = 0 and P_x <= 0
        S_x = 'C'
        make a connection with node x and i
    End
End
/* new connections of dominant individuals */
Get the node list max_connected_component contained in the max connected component of the
current network
For i in max_connected_component
    If S_i = 'C' and P_i >0
        candidate_node_list = camdidate_node_list.append(i)
    End
End
j = choice( candidate_node_list )
For x = 1 to N
    If d D_x >= 1 and S_x = 'C'
      and length( candidate_node_list )>0
        make a connection with node x and j
    End
End
/*Fermi update*/
k = 0.1
For x to N
    Select a random node y from the neighbor nodes of node x.
```

If $\dfrac{1}{1+\exp(-\dfrac{P_y - P_x}{k})} > random\_number$ // refer to Equation (6)

$S_x = S_y$

End

End

End
